# TDP-43 pathology in the basal forebrain and hypothalamus of patients with amyotrophic lateral sclerosis

**DOI:** 10.1186/s40478-014-0171-1

**Published:** 2014-12-24

**Authors:** Matthew D Cykowski, Hidehiro Takei, Paul E Schulz, Stanley H Appel, Suzanne Z Powell

**Affiliations:** Department of Pathology and Genomic Medicine, Houston Methodist Hospital, 6565 Fannin Street, Suite M227, Houston, TX 77030 USA; Houston Methodist Neurological Institute, Houston Methodist Hospital, Houston, TX USA; Department of Neurology, University of Texas Medical School at Houston, Houston, TX USA; Mischer Neuroscience Institute, Houston, TX USA; Department of Neurology, Houston Methodist Hospital, Houston, TX USA

**Keywords:** Amyotrophic lateral sclerosis, TDP-43, Basal forebrain, Hypothalamus

## Abstract

**Introduction:**

Amyotrophic lateral sclerosis is a neurodegenerative disease characterized clinically by motor symptoms including limb weakness, dysarthria, dysphagia, and respiratory compromise, and pathologically by inclusions of transactive response DNA-binding protein 43 kDa (TDP-43). Patients with amyotrophic lateral sclerosis also may demonstrate non-motor symptoms and signs of autonomic and energy dysfunction as hypermetabolism and weight loss that suggest the possibility of pathology in the forebrain, including hypothalamus. However, this region has received little investigation in amyotrophic lateral sclerosis. In this study, the frequency, topography, and clinical associations of TDP-43 inclusion pathology in the basal forebrain and hypothalamus were examined in 33 patients with amyotrophic lateral sclerosis: 25 men and 8 women; mean age at death of 62.7 years, median disease duration of 3.1 years (range of 1.3 to 9.8 years).

**Results:**

TDP-43 pathology was present in 11 patients (33.3%), including components in both basal forebrain (n= 10) and hypothalamus (n= 7). This pathology was associated with non-motor system TDP-43 pathology (Χ^2^= 17.5, p= 0.00003) and bulbar symptoms at onset (Χ^2^= 4.04, p= 0.044), but not age or disease duration. Furthermore, TDP-43 pathology in the lateral hypothalamic area was associated with reduced body mass index (W= 11, p= 0.023).

**Conclusions:**

This is the first systematic demonstration of pathologic involvement of the basal forebrain and hypothalamus in amyotrophic lateral sclerosis. Furthermore, the findings suggest that involvement of the basal forebrain and hypothalamus has significant phenotypic associations in amyotrophic lateral sclerosis, including site of symptom onset, as well as deficits in energy metabolism with loss of body mass index.

## Introduction

Amyotrophic lateral sclerosis (ALS) is a progressive neurodegenerative disease characterized by loss of upper and/or lower motor neurons, muscle wasting, and bulbar symptoms (e.g., dysarthria, dysphagia) [1]. The pathologic underpinnings of upper and lower motor neuron deficits in ALS include neuronal loss and gliosis in large neurons of “canonical” regions, including motor cortex, lamina IX of the ventral horn of the spinal cord, and somatic motor cranial nerve nuclei of the brainstem (XII, IX, and V) [2].

Detailed pathologic studies have also demonstrated surprisingly widespread, non-motor pathology in some ALS patients, typically in the form of ubiquinated cytoplasmic inclusions that are immunoreactive for transactivating responsive sequence (TAR) DNA-binding protein 43 kDa, or TDP-43 [3]. Pathologic TDP-43 deposition is presumed to induce neuronal dysfunction through the cytoplasmic accumulation of toxic C-terminal TDP-43 fragments, or alternately, via the loss of constitutively expressed nuclear TDP-43 that is critical in transcriptional regulation and RNA processing [4]. As such, widespread TDP-43 proteinopathy in “non-canonical” brain regions (striatum, amygdala, non-frontal cortex, hippocampus) may underlie multifocal neuronal dysfunction that contributes to complex non-motor phenotypes in ALS, including cognitive impairment with prominent frontal executive dysfunction [5] and extrapyramidal signs [1]. Clinicopathologic studies confirming this presumption are rare, but those that have been performed do support an association between extra-motor TDP-43 pathology and extra-motor clinical findings [6].

Somewhat unique among extra-motor features in ALS are alterations in energy metabolism [7], autonomic function [8], and in some patients [9,10] and animal models [11], involvement of the hypothalamic-pituitary axis. Deficits in energy metabolism are well recognized in ALS, with a paradoxical hypermetabolism occurring in many patients that combines with dysphagia to result in malnourishment and loss of body mass index (BMI) [12]. Along with advanced age and bulbar onset at diagnosis [13], loss of more than one BMI unit over two years is associated with a significantly shorter survival and more rapid disease progression [7]. Deficits in energy metabolism and autonomic function in ALS suggest the possibility of hypothalamic dysfunction, which may be indicated by pathologic TDP-43 deposition. This is further supported by evidence from Alzheimer disease [14], argyrophilic grain disease [15], Lewy body spectrum diseases [16], and multiple system atrophy [17], in which neurons of the hypothalamic region and adjacent basal forebrain are susceptible to neurodegenerative disease-associated cellular injury [18]. Although similar forebrain/hypothalamic pathology may be present in ALS and contribute to the extra-motor features of the disease [1,8], this has yet to be systematically investigated.

This study was carried out to determine whether the basal forebrain and hypothalamus are pathologically involved in ALS. We hypothesized that pathologic TDP-43 inclusions would be identified in a subset of ALS patients given the deficits in energy metabolism and autonomic function in ALS and frequent involvement of these regions in other neurodegenerative diseases. The frequency and topography of TDP-43 inclusions were examined in 33 ALS patients within these structures. Pathology in this region also was examined with respect to disease duration, the extent of TDP-43 proteinopathy, last available BMI, which is an indirect measure of energy metabolism, and patient age, since TDP-43 pathology may be seen in older patients without ALS [19].

## Materials and methods

Pathologic materials were reviewed for 56 consecutive patients with clinically diagnosed and pathologically confirmed ALS. Archival materials were screened for representative portions of basal forebrain and hypothalamus (defined further below). Thirty-three patients were selected for study based on the presence of appropriate anatomic regions.

### Autopsy and clinical data

Patient autopsies performed at the Houston Methodist Hospital included sections from all cerebral lobes, basal ganglia, thalamus, amygdala, hippocampus, midbrain, pons, medulla, cerebellum, spinal cord and pituitary. Immunostains performed on the majority of cases included tau (Dako, polyclonal, 1:40,000 dilution), beta-amyloid (Dako, clone 6 F/3D, 1:20 dilution), alpha-synuclein (EMD Millipore, clone AB5038, rabbit polyclonal, 1:4000), and TDP-43 (ProteinTech, 10782-2-AP, polyclonal antibody, 1:200). An immunostain for ubiquitin (Dako, polyclonal, 1:500 dilution) also was performed on several archived cases. Immunostaining was performed on brain tissue that was fixed in 20% formalin for a minimum of seven days following brain removal. Sections were further fixed in 10% formalin until processing and embedding. Formalin fixed paraffin embedded tissue was sectioned at 4 microns, mounted on plus coated slides and dried at 60°C. Immunostaining was performed on the BenchMark ULTRA^**©**^ platform (Ventana Medical Systems, Inc., Tucson, AZ) with appropriate positive and negative controls.

Neuropathologists at Houston Methodist Hospital (H.T., S.Z.P.) utilized established criteria to assess Alzheimer disease (AD) neuropathologic changes, including neurofibrillary changes [20,21] and parenchymal amyloid [22,23], Lewy body pathology [16], ALS pathology [24], cerebral amyloid angiopathy (CAA), and vascular brain injury (VBI). Clinical variables recorded included date of symptom onset and date of death (disease duration calculated as the difference between these), presenting symptoms, clinical dementia, and family history of ALS. Body mass index (BMI) was also recorded for patients where this data was available within ≤ 24 months of death (29 of 33 patients, median interval of 3.6 months between measurement and death, interquartile range 0.8-8.6 months). These studies were carried out with the approval of the Institutional Review Board at Houston Methodist Hospital (IRB-2-0114-0013).

### Identification of basal forebrain and hypothalamic components

Basal forebrain components were defined on H&E and TDP-43 stains and included ventral pallidum (VP), ventral striatum and small granule cell islands (VS/Is), the bed nucleus of the stria terminalis (BNST), lateral septal area (LSA), and other neurons of the substantia innominata (SI). VP included large pallidal neurons ventral to fibers of the anterior commissure [25]. SI included medium-sized neurons, which did not always respect anatomic borders, as well as magnocellular neurons (e.g., nucleus of the diagonal band, nucleus basalis) [26]. VS/Is included medium-sized striatal neurons and islands of small granule cells, located within or ventromedial to VS, as well as within SI and VP in the region of the olfactory tubercle [27]. Since the distinction between small granule cells comprising “Islands of Calleja” versus “interface” islands [28] is not clear, we adopted the descriptive term used here. BNST was defined as the wedge-shaped group of medium-sized neurons dorsal to anterior commissure, medial to the internal capsule, ventral to caudate nucleus, and lateral to the LSA [26,29].

Hypothalamic nuclear groups were defined on H&E and TDP-43 stains by cytologic characteristics and relative position using standard anatomic references [18,29-32]. Hypothalamic nuclei often merge with surrounding nuclear groups without distinct borders [29,30], due in part to inherent anatomic variability in the region [31]. Therefore, neuronal groups with similar positional and cytologic characteristics were grouped for recording of TDP-43 pathology. Nuclei examined at the chiasmatic level included discrete magnocellular neurosecretory neurons of the supraoptic (SON) and paraventricular (PaV) nuclei [30]. At the chiasmatic level, TDP-43 pathology in small- to medium-sized neurons of the preoptic area (POA) [31] (including “hypothalamic” or “chiasmatic gray” in some sources [18,30]) was recorded. At the tuberal and mammillary levels, neurons assessed included ventrally located, medium- to large-sized neurons of the medial mammillary nucleus, lateral tuberal nucleus (in some sections), and ventral portion of the tuberomammillary nucleus (MN/LTN/TMN). Likewise, large neurons lateral to the fornix and mammillothalamic tract were recorded as lateral hypothalamic area, which merges with the dorsal portion of the tuberomammillary nucleus (LHA/TMN) [29,32]. At the tuberal level, inclusions were also recorded in diffuse, small- to medium-sized neurons adjacent to the third ventricle, including those of the ventromedial hypothalamus (PVL/TBL/VMH). At the mammillary level, medial to fiber bundles of the mammillothalamic tract, medium- to large-sized periventricular neurons were recorded as posterior hypothalamic area (PHA) [31,32].

**Figure 1:**
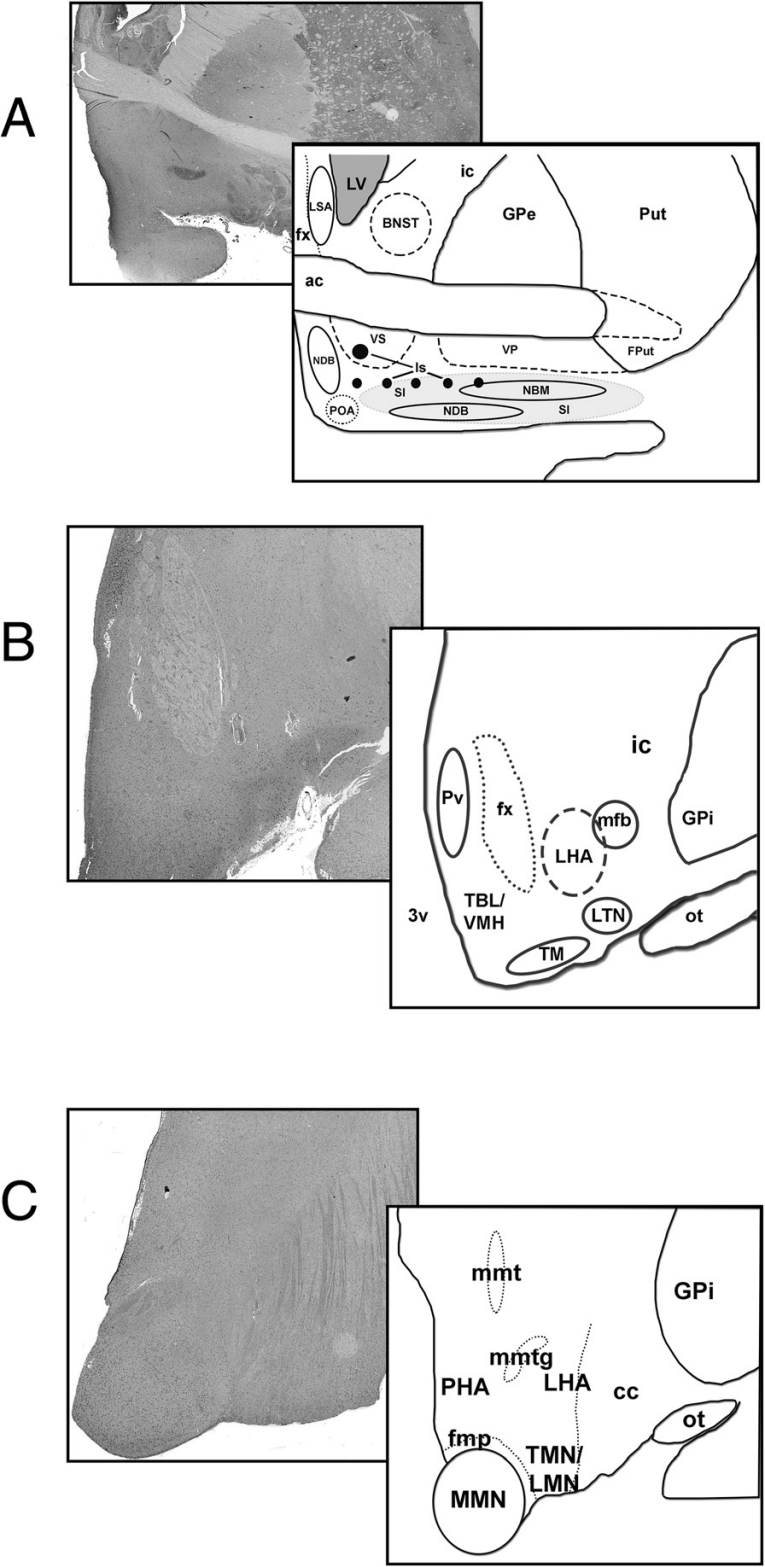
**Schematic demonstrating anatomic structures investigated in the basal forebrain (A), mid-hypothalamus (tuberal level) (B), and caudal hypothalamus (mammillary level) (C) (see** **Materials and methods** **for detail).** To the left of each schematic, grayscale renderings of TDP-43-stained sections of study patients are shown for comparison. In A, the continuity of rostral/chiasmatic hypothalamus and basal forebrain, as discussed in the text, is shown on both the histologic image (which also includes the optic chiasm) and on the schematic (POA). Abbreviations not defined in text: *ac* – anterior commissure, *cc* – crus cerebri, *fmp* – fasciculus mammillaris princeps (mammillary efferents)*, fx* – fornix, *GPe/i* – external/internal segments of the globus pallidus, *FPut* – fundus of the putamen, *ic* – internal capsule, *LV* – lateral ventricle, *mfb* - medial forebrain bundle, *mmt* – mammillothalamic tract, *mmtg* – mammillotegmental tract, *ot* – optic tract, *3v* – third ventricle.

These anatomic regions are shown in schematic form for basal forebrain (Figure 1A), including LSA and BNST, and hypothalamus at the caudal chiasmatic/tuberal level (Figure 1B), and level of the mammillary bodies (Figure 1C).

### Assessment of TDP-43 pathology

Two authors (M.D.C., H.T.) independently rated TDP-43 pathology in the aforementioned areas. The location of pathologic TDP-43 inclusions was recorded and classified by morphologic sub-type as follows: neuronal cytoplasmic (NCI), neuronal intranuclear (NNI), glial cytoplasmic (GCI), and dystrophic neurites. For cases with TDP-43 inclusion pathology, quantitative measures were performed in the area of greatest hypothalamic pathology (or basal forebrain in cases without hypothalamic pathology). For these cases, any cellular TDP-43 inclusions (NCI, GCI) were recorded within three consecutive high-power microscopic fields (HPF) at 400 × magnification (0.19625 mm^2^ per HPF). Global TDP-43 pathologic burden was classified as canonical (brainstem, spinal cord, motor cortex) or non-canonical (canonical regions *plus* dorsal striatum, thalamus, non-frontal isocortex, and mesial temporal lobe).

### Statistical analysis

Two-sided Mann–Whitney (Wilcoxon rank sum) testing was used to determine whether: (a) disease duration significantly differed between patients with and without basal forebrain/hypothalamic pathology, (b) disease duration significantly differed between patients with canonical and non-canonical TDP-43 pathology, (c) BMI significantly differed in patients with and without basal forebrain/hypothalamic pathology, (d) BMI significantly differed in patients with and without LHA pathology, and (e) age at death significantly differed between those with and without basal forebrain/hypothalamic TDP-43 pathology. Chi-squared (Χ^2^) testing was used to determine whether basal forebrain/hypothalamic pathology was associated with (a) non-canonical TDP-43 pathology and (b) bulbar/respiratory onset.

## Results

### Clinical characteristics and autopsy findings

Table 1 lists demographic information for 33 ALS patients. Presenting symptoms included unilateral extremity weakness with or without muscle fasciculations (46.4%), bulbar symptoms such as slurred speech, shortness of breath, dysarthria, and difficulty swallowing (25.0%), muscle cramps (17.8%), bilateral extremity or generalized weakness with/without fasciculations (17.8%), falls (10.7%), and foot drop (3.5%). One patient showed clinically evident cognitive impairment with episodes of disorientation and word-finding difficulties. In two patients with a family history of ALS the genes implicated were not known (one of the two had *SOD1*, *TARDBP*, *ANG*, *FUS*, *FIG4* studied) though both patients had ALS TDP-43 pathology outside of the hypothalamic region.

**Table 1:** **Demographics and non-ALS pathology in 33 patients**

Average age at death (years)	62.7 (s = 9.09)
Male/Female	n = 25/n = 8
Median duration (years)	3.1 (interquartile range, 2.2-4.8)
Brain weight (grams)	1348.6 (s = 156.97)
Any neurofibrillary pathology	n = 24
Any parenchymal amyloid	n = 9
Thal stage ≥ II	n = 7
Braak stage ≥ III^1^	n = 4
CERAD ≥ “moderate”	n = 2
CAA present^2^	n = 6
VBI present^3^	n = 3
LB pathology	n = 0

*Abbreviations:* *CAA* cerebral amyloid angiopathy, *CERAD* Consortium to Establish a Registry for Alzheimer Disease (see text), *LB* Lewy body, *VBI* vascular brain injury.

Notes: ^1^One patient with Braak stage III had co-existing argyrophilic grain disease (AGD). ^2^CAA was diffuse in only 1 of these 6 patients. ^3^VBI was present in one patient each as remote infarct (left temporal lobe), hippocampal microinfarct, and acute/subacute hemispheric infarct.

At autopsy, all patients had pathologically confirmed ALS. Table 1 lists non-ALS brain pathologies identified, including AD neuropathologic changes, CAA and VBI.

### TDP-43 inclusion pathology in basal forebrain and hypothalamus

**Figure 2:**
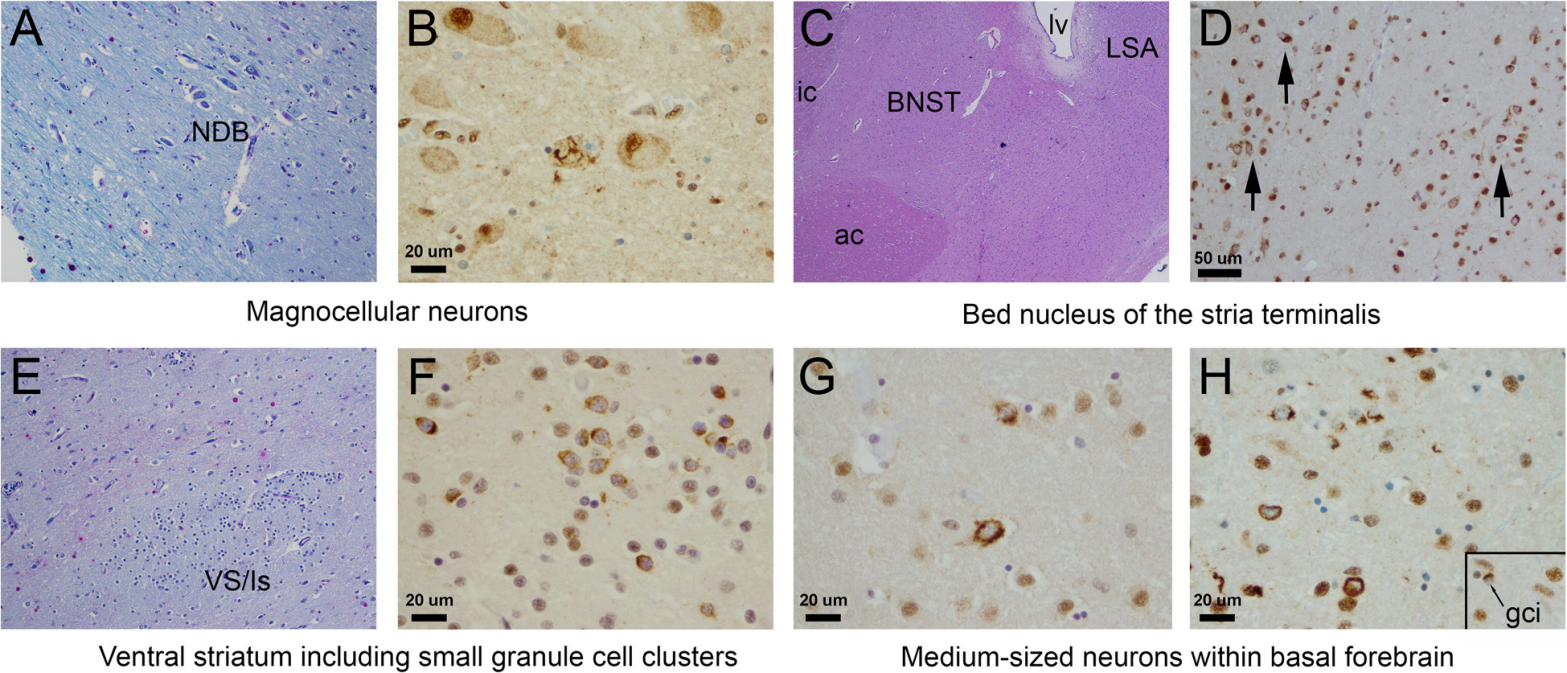
**Basal forebrain pathology in ALS including TDP-43 immunostain examples from six different ALS patients. (A)** Representative low-power histologic section (LFB/PAS-hematoxylin) demonstrating magnocellular neurons within nucleus of the diagnonal band (NDB) (pial surface at bottom left of image). **(B)** Skein-like TDP-43 inclusion within the horizontal limb of the NDB (400 ×, scale bar is 20 microns). **(C)** Representative lower-power histologic section demonstrating the location of the bed nucleus of the stria terminalis (BNST) and adjacent lateral septal area (LSA) at the level of the internal capsule (ic), anterior commissure (ac), and lateral ventricle (lv) (compare to Figure 1A). **(D)** Numerous NCIs within the BNST (highlighted by black arrows) (200 ×, scale bar is 50 microns). **(E)** Low-power LFB/PAS-H histologic section demonstrating ventral striatum (Vs/Is) including clusters or “islands” of small neurons (bottom center of image). **(F)** Numerous NCIs in small neurons of the VS/Is (400 ×). **(G,H)** Medium-sized neurons of “substantia innominata” with frequent TDP-43 NCIs and neurities (bottom left of H). These cells could not be attributed to a specific nuclear group or region on H&E stain (see text) (400 ×) (H, inset). A glial cytoplasmic inclusion (at 400 ×) is shown for comparison of cell size with small- and medium-sized neurons with NCIs in panels D, F, G, and H.

**Figure 3:**
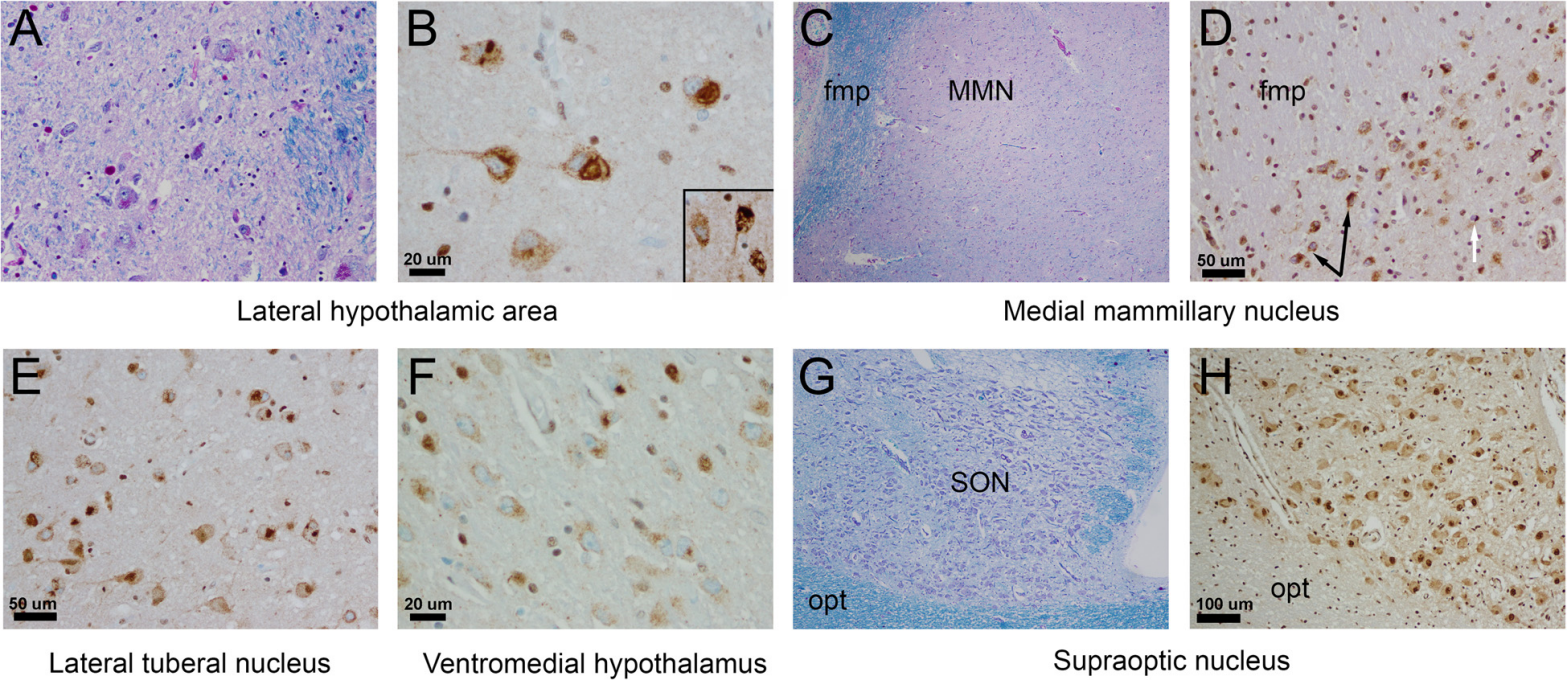
**Hypothalamic pathology in ALS including TDP-43 immunostain examples from six different ALS patients (five pathologic, one normal). (A)** Representative LFB/PAS-hematoxylin-stained section at intermediate magnification demonstrating large neurons and myelinated fiber bundle within the lateral hypothalamic area (LHA). **(B)** NCIs in the LHA of two different patients (both at 400 ×, scale bar is 20 microns), including a patient with dense, filamentous inclusions (inset). **(C)** Representative LFB/PAS-hematoxylin-stained section at low power magnification demonstrating the medial mammillary nucleus (MMN) of the mammillary body and the fasciculus mammillaris princeps (fmp), representing the common origin of the mammillotegmental and mammillothalamic tracts. **(D)** Numerous NCIs (black arrows) and rare GCIs (white arrow) within the MMN (200 ×, scale bar is 50 microns). **(E)** NCIs within the lateral tuberal nucleus (200 ×) and NCIs, including many granular preinclusions, within ventromedial hypothalamus at the tuberal level (400 ×) **(F)**. **(G)** Characteristic low power magnification appearance on LFB/PAS-hematoxylin-stained section of large, loosely cohesive neurons in the supraoptic nucleus (SON) overlying the optic tract (opt). **(H)** SON neurons (seen here at 100 ×, scale bar is 100 microns) did not demonstrate TDP-43 pathology and inclusions were likewise very infrequent in the large, secretory neurons of the paraventricular nucleus (not shown).

Pathology in basal forebrain and hypothalamus was present in 11 of 33 cases (33.3%). This included components of basal forebrain in 10 patients: VS/Is, n = 9 of 26 patients with the structure present; BNSTL, n = 8 of 21; SI neurons, n = 6 of 28; typically in medium-sized neurons with magnocellular neurons having filamentous/skein-like inclusions in only two cases; LSA, n = 2 of 15; and, VP, n = 1 of 21. This also included components of the hypothalamus in 7 patients, including LHA/TMH, n = 5 of 28; PHA, n = 3 of 24; MB/LTN/TMN, n = 6 of 19; PVN/POA, n = 2 of 16; and PVL/TBL/VMH, n = 4 of 10. The SON was not involved (n = 13). Representative findings are demonstrated for basal forebrain and hypothalamus in Figures 2 and 3, respectively.

Neuronal inclusions were uniformly cytoplasmic (NCIs), while intranuclear inclusions were not identified. The density of TDP-43 cellular inclusions varied between 1/three HPFs to 72/three HPFs: the average number of inclusions was 8.42 (sd = 18.75), with a per field average of 2.8 inclusions. NCI morphology consisted of granular preinclusions (e.g., Figure 3F), dense perinuclear and cytoplasmic inclusions that were crescent-shaped or globoid (e.g., Figure 2D and F, Figure 3B and E), and rarely, densely staining filamentous and skein-like inclusions (Figure 2B and inset of Figure 3B). GCIs were frequently identified in patients with NCIs in basal forebrain and hypothalamus. GCIs could be distinguished from small neurons with NCIs in the forebrain on the basis of cell size (see inset of Figure 2H for comparison). Thick, tortuous TDP-43 positive neurites were occasionally seen (see bottom left of Figure 2H) but were uncommon.

For all cases positive for TDP-43 pathology in the regions under investigation (n = 11), two authors (MDC, SZP) performed a post-hoc rating of gliosis (on H&E stain) as absent, mild, moderate, or severe. One patient had mild gliosis in the preoptic area of the hypothalamus and this same patient had mild to focally moderate gliosis in basal forebrain (most apparent in the BNST). Three additional patients had mild gliosis in basal forebrain only, typically most apparent medially including within BNST. A fifth patient had moderate gliosis more diffusely throughout basal forebrain ventral to the anterior commissure (this patient had 63 TDP-43 cellular inclusions/three HPFs). Neuronal loss was not evident on H&E stain in these patients.

### Extent of TDP-43 pathology

ALS pathology in typical (canonical) regions was characterized by neuronal loss and gliosis with ubiquitinated or TDP-43 immunoreactive inclusions. Brain regions implicated included spinal cord (n = 33), brainstem motor nuclei (n = 27), other brainstem nuclei (n = 17), and agranular frontal cortex (n = 22). Seventeen of 33 patients had pathologic involvement *limited* to these canonical regions. The remaining 16 patients had pathology in these regions *plus* non-canonical pathology, i.e. additional involvement of hippocampus (n = 11), amygdala (n = 11), non-frontal isocortex (n = 9), dorsal striatum (n = 10), and thalamus (n = 8).

### Association of pathology in basal forebrain/hypothalamus with non-canonical pathology

TDP-43 pathology in basal forebrain/hypothalamus was seen only with non-canonical pathology (Χ^2^ = 17.5, p = 0.00003). Moreover, all but one case with hypothalamic TDP-43 pathology occurred with concurrent basal forebrain pathology. In contrast, sparse basal forebrain pathology (range of 2–3 NCIs in three HPFs) occurred in three cases without hypothalamic pathology. An additional case with dense basal forebrain pathology (63 NCIs in three HPFs) did not have hypothalamic tissue available for study.

### Association of pathology with clinical presentation

There was a significant association between bulbar onset symptoms and basal forebrain/hypothalamic pathology (Χ^2^ = 4.04, p = 0.044) with 71.4% of all patients having bulbar symptoms at disease onset demonstrating this pathology (compared to 28.6% of patients without bulbar symptoms at onset).

### Association of pathology with disease duration and patient age

For patients with complete data on disease duration (n = 30), this parameter did not significantly differ between patients with basal forebrain/hypothalamic TDP-43 pathology (median of 3.26 years, interquartile range, 2.5-5.3) and without (median of 3.1 years, interquartile range, 2.1-3.5) (W = 90, p = 0.55). Likewise, the density of TDP-43 inclusions was not correlated to disease duration (Spearman rho = 0.18, p = 0.33). Disease duration also did not significantly differ between patients with non-canonical (median of 3.0 years, interquartile range, 2.4-4.7) and canonical TDP-43 pathology (median of 3.1 years, interquartile range, 2.1-4.9) (W = 109, p = 0.92). Patient age at death did not significantly differ between those with (mean = 64.1 years, sd = 11.33 years) and without (mean = 62.0 years, sd = 7.96 years) TDP-43 pathology in basal forebrain/hypothalamus (W = 95, p = 0.33).

### Association of basal forebrain and hypothalamic pathology with BMI

**Figure 4:**
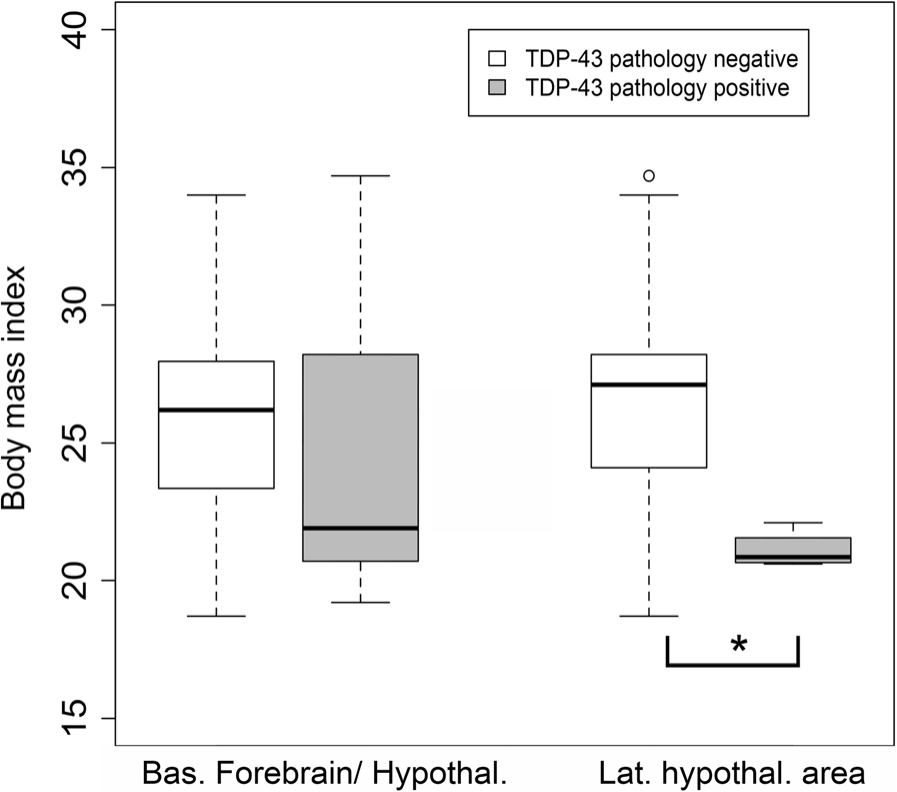
**Boxplots of BMI relative to involvement of any region in the basal forebrain and/or hypothalamus (left) versus LHA (right) for patients with a BMI measure within** ≤ **24 months the time of death (median interval of 3.6 months).** Plots represent BMI for basal forebrain/hypothalamus (n = 29; 19 patients without TDP-43 pathology and 10 with inclusion pathology) and for patients with the LHA sampled (n = 25; 21 patients without TDP-43 pathology and 4 with inclusion pathology). There was no significant difference in BMI for patients with and without TDP-43 pathology in any component of forebrain/hypothalamus (left). For the LHA, a significant difference in BMI was identified between patients with and without TDP-43 pathology (p = 0.02). Boxplot prepared in R (R Foundation for Statistical Computing, Vienna, Austria, 2014).

BMI did not significantly differ between patients with (median BMI of 21.9 kg/m^2^, interquartile range, 20.8-28.2) and without (median of 26.2 kg/m^2^, interquartile range, 23.4-28.0) any basal forebrain and/or hypothalamic pathology (W = 82, p = 0.57). BMI did significantly differ, however, between patients with (median of 20.9 kg/m^2^, interquartile range, 20.7-21.3) and without (median of 27.1 kg/m^2^, interquartile range, 24.1-28.2) pathology in the LHA specifically (W = 11, p = 0.023) (see Figure 4). Both initial BMI (at a median interval of 8.9 months from symptom onset) and last recorded BMI (3.6 months from measurement until patient death, see Materials and methods for detail) were available in 25 patients. The percent differences between initial and last recorded BMI (positive percent difference values indicating loss of BMI points) were 6.0%, 8.3%, and 26.6% for patients with no forebrain/hypothalamic TDP-43 pathology, *any* forebrain/hypothalamic pathology, and LHA/TMN region TDP-43 pathology, respectively.

## Discussion

Patients with ALS may experience a variety of extra-motor symptoms that have been referred to as “ALS-plus” syndromes, but that may simply represent a more complete spectrum of the disease [1]. These signs and symptoms include energy metabolism deficits [12], subclinical autonomic nervous system dysfunction [8], and frontal executive dysfunction manifesting as cognitive impairment in up to 51% of patients [5].

**Figure 5:**
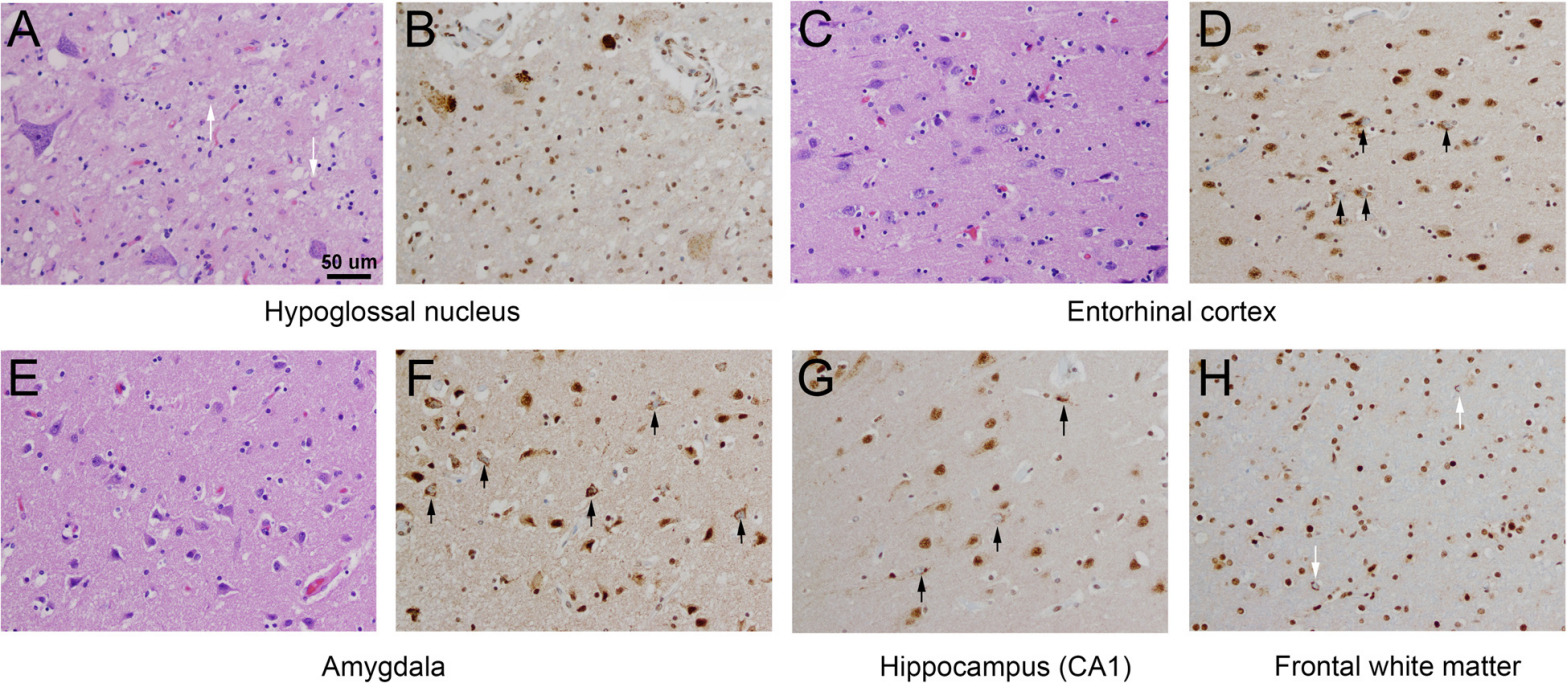
**Canonical and non-canonical pathology outside forebrain/hypothalamus in an ALS patient with forebrain/hypothalamic TDP-43 pathology, bulbar symptom onset, and survival of 29.8 months.** Pathology in canonical regions, such as lower motor neurons of the hypoglossal nucleus **(A)**, was characterized by neuronal loss, gliosis (white arrows), rarefaction of neuropil, and on TDP-43 immunostain **(B)**, pleomorphic inclusions. Non-canonical TDP-43 pathology was seen in the deep layers of entorhinal cortex (**C, D;** black arrows indicate neuronal cytoplasmic inclusions in all panels), basolateral amygdala immediately overlying the temporal horn of the lateral ventricle **(E, F)**, and hippocampal sector CA1 **(G)**. This patient also demonstrated glial cytoplasmic inclusions (**H**, white arrows). All images taken at 200× (scale bar of 50 microns as shown in panel A applies to all panels).

This study is the first to systematically investigate whether TDP-43 pathology in the basal forebrain and hypothalamus of ALS patients might contribute to these extra-motor symptoms, given the important roles of these regions in cognition, energy metabolism and thermoregulation, endocrine function, feeding behaviors, and autonomic responses [25,33,34]. TDP-43 pathology was identified in this region in one-third of patients and stereotypic involvement of certain structures was found (e.g., VS/Is, BNST, medium-sized neurons of SI, LHA/TMN, MB/LTN) with little or no involvement of other structures (VP, SON, PVN). Pathology in this region was seen only in the context of other non-canonical TDP-43, including within dorsal striatum, thalamus, non-frontal cortex, hippocampus (each of these regions involved in seven patients) and amygdala (in eight patients) (see Figure 5). No patients with isolated motor neuron and hypothalamic pathology were identified, and with a single exception, hypothalamic pathology appeared to follow basal forebrain pathology. As such, the pathology identified here is best placed in context of the progressive, widespread TDP-43 proteinopathy recently characterized as stage III and stage IV for ALS TDP-43 pathology [35].

We also tested whether AD neuropathology [36] or aging [19] influenced the TDP-43 pathology identified. The lack of advanced AD neuropathology (see Table 1) and demonstration that age did not influence TDP-43 pathology in the cohort suggests this is not the case and that these findings are in indeed within the spectrum of ALS pathology.

### The location of forebrain and hypothalamic pathology in ALS

The finding of basal forebrain and hypothalamic pathology in ALS patients is consistent with earlier observations. One recent study of pathologic staging in ALS identified the heaviest burden of subcortical TDP-43 pathology in the VS [35]. We similarly identified NCIs in VS, but also within the associated small granule cell islands adjacent to VS and within the area of the olfactory tubercle (Figure 2E, F). Though their role is poorly understood, these small neurons are thought to receive afferent input from serotonergic and catecholaminergic cells of the brainstem and may have projections to dendrites of adjacent striatal cells [37]. NCIs also were identified in the LSA (two patients) with both of these patients having filamentous or skein-like inclusions in magnocellular neurons of basal forebrain (nucleus of the diagonal band). This relationship is intriguing as a “hippocampal-septal area-diagonal band” circuit has been proposed as a structural subunit of basal forebrain organization [33] and raises the question of whether the pathology identified may reflect underlying structural and functional homologies between neuronal groups.

TDP-43 pathology was also identified in medium-sized, nondescript neurons of the SI that could *not* be attributed to VP, VS/Is, or magnocellular neuron groups (see Figure 2H). The possibility that these cells represent extensions of amygdala into the basal forebrain [33] would be consistent with the frequent involvement of amygdala in ALS patients, as well as with frequent concurrent pathologic involvement of the BNST (also identified here), which itself is a portion of the “extended” amygdala [38]. TDP-43 pathology in amygdala and extended amygdala may in part underlie behavioral alterations in some ALS patients that are usually present in non-ALS patients with bilateral amygdala damage [39].

There are fewer examples of pathologic involvement of the hypothalamus in the ALS literature. Takahashi and colleagues reported a single case of ALS with cell loss and gliosis in cortical and subcortical neurons, including within the MB [40]. A recent study from Mayo Clinic Jacksonville identified TDP-43 NCIs in the hypothalamus of ALS and FTLD/ALS patients (more frequently in the latter group) [41]. We identified neuronal cytoplasmic TDP-43 inclusions in five patients, most frequently within neurons of the LHA (see Figure 3B), as well as within neurons of MB and adjacent tuberal nuclei. Neurons of hypothalamus, particularly the LHA, may receive both neural and neurohormonal input to regulate a number of functions including metabolic rate, feeding behavior, and autonomic function [34]. Further studies are also warranted to examine affected nuclei using both TDP-43 and proteins marking neuronal sub-populations (e.g., orexin/hypocretin, alpha-melanocyte stimulating hormone).

### Potential implications of forebrain and hypothalamic pathology in ALS

The finding of significantly reduced BMI in patients with LHA pathology is intriguing (see Figures 3 and 4) and suggests the possibility that pathology in this region may contribute to metabolic disturbances and weight loss in ALS, in addition to increased muscle work, possible mitochondrial defects [12], and dysphagia/bulbar symptoms [42]. Patients with ALS have a paradoxical elevation in resting energy expenditure (~10% greater than matched controls), which is surprising given their loss of fat-free mass (itself an important determinant of basal metabolic rate) [43]. The pathophysiologic bases for this finding remain unexplained but may relate to nutritional status, which is prognostically important [13]. However, several caveats require mention. First, rare patients *without* forebrain and hypothalamic pathology had a relatively low last available BMI (see Figure 4), which indicates the mechanisms underlying metabolic alterations and BMI loss in ALS are multiple and not entirely understood. Second, a significant association of bulbar onset of symptoms and forebrain/hypothalamic pathology was identified. Even limb-onset patients often experience bulbar symptoms late in their disease course. Aggressive clinical interventions are performed in such patients to counteract the detrimental effects of dysphagia on BMI [42]; still, the exact contributions of pathologic and clinical factors on weight loss are difficult to estimate. An additional question is whether BMI changes in ALS patients are the result of extensive TDP-43 proteinopathy or the result of neuronal dysfunction in specific populations. We had identified forebrain and hypothalamic pathology only in patients with widespread TDP-43 inclusions (e.g., Figure 5). BMI did not significantly differ when *any* forebrain/hypothalamic pathology was considered (Figure 4, left box plots), which suggests the LHA region pathology may be important in this respect. However, this does not exclude the possibility that TDP-43 pathology in other brain regions influences metabolic rate and BMI in ALS.

There likely are several other clinical implications for patients with hypothalamic pathology in ALS since this structure subserves affective behaviors, autonomic function, sleep-wake cycles, thermoregulation, feeding and reproductive behaviors, water balance, and endocrine function [29-31]. Clinical studies of hypothalamic function in ALS are rare so the clinical correlates of the pathology are difficult to estimate. Nonetheless, reduced growth hormone has been reported in up to 52% of ALS patients [10]. Likewise, inappropriate secretion of antidiuretic hormone has also been documented [9], which raises the possibility of hypothalamic neuronal dysfunction (e.g., osmoreceptors). Significant pathology in the LHA is also likely to impair the ascending arousal system in ALS patients. LHA and forebrain nuclei receive afferents from monoamenergic neurons in the brainstem (e.g., locus ceruleus), among other sites, and have a profound influence on wakefulness via projections to cerebral cortex [44]. Ultimately, any anatomic focus of significant hypothalamic pathology is likely to have *multiple* effects given the integrative role of this structure [31]. This is reflected in widespread connections to hippocampus, corticomedial and basolateral amygdala, basal ganglia, and midbrain tegmentum and reticular formation [29,31,32], as well as thalamus, dorsal medulla, neurohypophysis, preganglionic neurons of spinal cord [29], and isocortex [31,32,34]. The implications of TDP-43 pathology in this region are further complicated by the fact that basal forebrain and hypothalamic neurons are in anatomic contiguity (e.g., septal area, diagnonal band, and rostral hypothalamus) [30], are interconnected (e.g., via medial forebrain bundle), and for hypothalamus, have internuclear short fiber connections [32].

### Extent of TDP-43 pathology and disease duration in ALS

An important finding of this study is an asynchrony between disease extent and clinically determined disease duration. Similar results have been observed in other autopsy series [35] and in reports of patients with rapid disease progression yet widespread TDP-43 pathology [45]. These findings support a model of non-linear propagation of neuropathology in this disease that is not time-dependent [46]. As such, pathology in ALS appears distinct from AD and Lewy body spectrum-diseases, in which extent and intensity of the tauopathy and alpha-synucleinopathy, respectively, are presumed to be modified by disease duration with a spectrum of pre-clinical pathologic stages [16,20].

Three patients in this study, for example, had unusually long survival (>6.5 years), and their pathology was limited to canonical regions (spinal cord, brainstem, agranular frontal cortex). Conversely, three different patients with disease durations less than 2.5 years had extensive TDP-43 proteinopathy, including within the regions investigated in this study. The determinants of extent of TDP-43 pathology, of which disease duration does not appear to be one, therefore remain unclear. Age has been suggested as a negative prognostic factor in ALS, but our analysis shows this was not a determinant of the extent of TDP-43 pathology. Immune dysregulation may play a role as patients with “rapidly progressive” ALS have a reduction in regulatory T-lymphocytes and reduction in transcription factors required for the function of this T-cell population (i.e., FoxP3) [47]. This suggests the intriguing possibility that short-lived patients with immune dysregulation may be among those with widespread TDP-43 deposition and rapid progression. Lastly, the significant association of basal forebrain and hypothalamic pathology with bulbar symptoms at onset identified here suggests the possibility that the site of disease initiation may play an important role in the propagation and final extent of disease. These and other factors that influence the kinetics and extent of TDP-43 spread are poorly understood and are deserving of further investigation.

### Study limitations

There are limitations of the present study due to the retrospective design. First, the study was conducted with archived sections available or with supplemental sections that could be generated from stored tissues. In rare instances, involving older archived cases, one or more anatomic regions were not available in the original material and no additional tissue was available for sampling. Prospective sampling in future ALS cases will ensure more uniformity in the anatomic regions available for assessment. Based on our findings, two sections are likely to yield the most valuable pathologic data for future studies. The first, inclusive of basal forebrain, could be taken at level of the optic chiasm, anterior commissure, septal area, and BNST, and would demonstrate LSA, BNST, VS/Is, VP, and rostral hypothalamus (Figure 1A). The second, inclusive of tuberal and caudal hypothalamus, could be taken at the level of the tuber cinereum and fornix and would demonstrate LHA/TMN, portions of MB, LTN, periventricular nuclei of the tuberal region including VMH, and the PHA (Figure 1B). Standardized sections across many non-ALS and ALS patients would also allow for more precise stereological analysis to determine the extent of neuronal loss, which is difficult to estimate by H&E and TDP-43 stains.

The ability to determine the clinical sequelae of the pathology described herein is also limited by the retrospective nature of the study. Future clinicopathologic studies of these clinical features with respect to the brain regions identified here will further clarify the importance of these pathologic findings in the extra-motor manifestations of ALS. This is particularly true with respect to measures of energy metabolism. The optimal clinical measures would include careful documentation of BMI at multiple time points [7] and measured resting energy expenditure [42] as calculated resting energy expenditure underestimates the hypermetabolism observed in ALS patients [43]. The pathologic findings documented herein provide a basis for the closer examination of these clinical measures with respect to TDP-43 pathology.

## Conclusions

This study has demonstrated that a subset of patients with ALS have TDP-43 pathology within basal forebrain and hypothalamus, which may contribute to the extra-motor symptoms of the disease, including alterations in energy metabolism, autonomic nervous system dysfunction, and frontal executive dysfunction. The pathologic involvement was fairly stereotypic, and in nearly all cases pathologic involvement of forebrain components (VS/Is, BNST, medium-sized neurons of SI) preceded hypothalamic involvement (LHA/TMN, MB/LTN). Basal forebrain and hypothalamic pathology was associated with the presence of other non-canonical TDP-43 inclusion pathology but was not associated with disease duration. Finally, the presence of forebrain/hypothalamic pathology was associated with bulbar onset of disease, which suggests the possibility of a unique and previously unrecognized clinico-pathological relationship in ALS.

### Ethical approval

All procedures performed in studies involving human participants were in accordance with the ethical standards of the institutional and/or national research committee and with the 1964 Helsinki declaration and its later amendments or comparable ethical standards.

### Consent

Written consent was obtained by the patients and/or their representatives to allow for their autopsy tissue to be used for research purposes. The study was also carried out with IRB approval from Houston Methodist Hospital.
